# Endocrinopathies after Allogeneic and Autologous Transplantation of Hematopoietic Stem Cells

**DOI:** 10.1155/2014/282147

**Published:** 2014-04-30

**Authors:** Francesco Orio, Giovanna Muscogiuri, Stefano Palomba, Bianca Serio, Mariarosaria Sessa, Valentina Giudice, Idalucia Ferrara, Libuse Tauchmanovà, Annamaria Colao, Carmine Selleri

**Affiliations:** ^1^Department of Sports Science and Wellness, University “Parthenope” Naples, 80133 Naples, Italy; ^2^Sterile Techniques SSD, AOU “S. Giovanni di Dio e Ruggi d'Aragona” Salerno, Salerno, Italy; ^3^Department of Clinical Medicine and Surgery, University Federico II Naples, 80131 Naples, Italy; ^4^Unit of Obstetrics and Gynaecology, “Arcispedale Santa Maria Nuova”, IRCCS, 42123 Reggio Emilia, Italy; ^5^Hematology and Hematopoietic Stem Cell Transplant Center, Department of Medicine and Surgery, University of Salerno, 84131 Salerno, Italy

## Abstract

Early and late endocrine disorders are among the most common complications in survivors after hematopoietic allogeneic- (allo-) and autologous- (auto-) stem cell transplant (HSCT). This review summarizes main endocrine disorders reported in literature and observed in our center as consequence of auto- and allo-HSCT and outlines current options for their management. Gonadal impairment has been found early in approximately two-thirds of auto- and allo-HSCT patients: 90–99% of women and 60–90% of men. Dysfunctions of the hypothalamus-pituitary-growth hormone/insulin growth factor-I axis, hypothalamus-pituitary-thyroid axis, and hypothalamus-pituitary-adrenal axis were documented as later complicances, occurring in about 10, 30, and 40–50% of transplanted patients, respectively. Moreover, overt or subclinical thyroid complications (including persistent low-T3 syndrome, chronic thyroiditis, subclinical hypo- or hyperthyroidism, and thyroid carcinoma), gonadal failure, and adrenal insufficiency may persist many years after HSCT. Our analysis further provides evidence that main recognized risk factors for endocrine complications after HSCT are the underlying disease, previous pretransplant therapies, the age at HSCT, gender, total body irradiation, posttransplant derangement of immune system, and in the allogeneic setting, the presence of graft-versus-host disease requiring prolonged steroid treatment. Early identification of endocrine complications can greatly improve the quality of life of long-term survivors after HSCT.

## 1. Introduction

Autologous- (auto-) and allogeneic- (allo-) hematopoietic stem cell transplant (HSCT) programs started in Europe about five decades ago and today their cumulative number exceeds 30.000 per year. Main underlying diseases leading to auto- and allo-HSCT are acute leukemias and myelodysplastic syndromes in the allogeneic setting and multiple myelomas, lymphomas, and leukemias in the autologous setting [1].

The progressive increase in the number of auto- and allo-HSCT performed in the last four decades for the treatment of malignant and nonmalignant hematological diseases has been accompanied by a parallel significant increase of long-term survivors due to a progressive decrease of transplant-related mortality. Given this progressive increase in the number of long-term survivors after auto- and allo-HSCT, ever greater attention by physicians is nowadays paid to prevention and to early diagnosis of early and late complications of HSCT procedure which may worsen the quality of life of transplant recipients [1–3].

The endocrine system is one of the most frequent targets of post-HSCT complications. However, the majority of the data available on the early and late effects of HSCT on the endocrine system refers to the pediatric population, while still few are the prospective studies on adult transplanted patients. The underlying diseases, previous pretransplant therapies, the age at HSCT, the use of total body irradiation (TBI), its cumulative dose and administration schedule, and posttransplant treatments are the main risk factors for endocrine complications after HSCT [4, 5]. Both auto- and allo-HSCT procedures are preceded by conditioning regimens consisting of high-dose antiblastic treatments, associated or not with TBI; this is aimed at eradicating the underlying hematologic disease (mostly a malignancy) and suppression of the host immune system in the allogeneic setting. Most of the data concerning the functioning of the endocrine system is derived from the study of patients who underwent TBI as a part of pretransplantation treatments [6]. TBI has been reported to be responsible for a large part of posttransplant endocrinopathies. Only a few studies have been carried out in adult HSCT patients treated without TBI, so the impact of chemotherapy alone and/or of other possible risk factors on the endocrine system dysfunction has not been completely established in adult HSCT recipients [7, 8].

Allo-HSCT is associated with a more severe derangement of the immune system than auto-HSCT, due to the severe immunosuppressive effect of the conditioning regimens to avoid graft rejection, the cytokine storm occurring at time of transplantation, the prolonged administration of multiple immunosuppressive drugs to prevent graft-versus-host disease (GVHD), and the frequent development of acute or chronic GVHD resulting in additional relevant alterations of the immune system. Nevertheless, auto-SCT also is associated with a marked immunosuppression due to highly aggressive lympho- and myeloablative conditioning regimens that are likely followed by a resetting of immune system. Therefore, autologous HSCT has had an emerging role in the last decades as a promising treatment for several autoimmune diseases [9–11].

The aim of this review is to describe the most commonly observed endocrine complications after HSCT in our center, by integrating the experience of international scientific literature. Our experience is based on managing main endocrine dysfunctions in more than 300 long-term survivors after auto- (*n* = 228) and allo-HSCT (*n* = 120) up to 15 years after transplant.

## 2. Hypothalamic-Pituitary-Gonadal Axis in Women and Men

### 2.1. Women

Ovarian failure is the most frequent complication after auto- and allo-HSCT. It has been mainly considered a direct consequence of radiation therapy and high-dose chemotherapy. Posttransplant recovery of the ovarian function is rare and appears to be age-dependent, occurring more frequently in young girls than in adult women [4, 12–15]. It has been calculated that, with increasing of age at transplantation, the probability of recovery of ovarian function declines according to coefficient of 0.8 per year [16]. Although the effect of cytotoxic agents and TBI on the ovary is dose-dependent [16–19], the radiation dose responsible for the death of 50% of human oocytes (LD50) was estimated to be less than 4 Gy [20]. Also almost all antineoplastic drugs, and especially alkylating agents, exert toxic effects on the ovaries in a dose-dependent way; this is linked not only to a direct damage of oocytes but also to damage of the supporting granulosa cells of both proliferating and quiescent follicles [21, 22]. The known risk factors for gonadal damage after HSCT are summarized in Table 1.

In a large study of 144 women transplanted for leukemia after conditioning with cyclophosphamide and TBI, Sanders et al. reported the occurrence of hypergonadotropic amenorrhea in 100% of the women in the first years after SCT, with a delayed recovery of ovarian function in only 6% of cases [18]. In women receiving as conditioning regimen only chemotherapy with cyclophosphamide (CY), ovarian function recovered in 31% of patients transplanted at age <26 years but in none of those over this age [16]. The addition of busulfan (BU: 16 mg/kg) to low or high doses of cyclophosphamide (CY: 120 and 200 mg/kg, resp.) caused permanent ovarian damage in everyone, with exception of a few cases [23–25]. Chatterjee et al., who investigated the acute effects of high-dose polychemotherapy with or without TBI on ovarian function in a large cohort of transplanted women, claim that the severity of acute injury is predictive of the probability of the later recovery of ovarian cycles [25].

In our experience, all women except one had experienced an ovarian insufficiency, regardless of the type of HSCT (auto- or allo-HSCT). Ovarian insufficiency manifested as secondary amenorrhea associated with hypergonadatrope hypogonadism and reduced volumes of ovaries and uterus. About one-third of women went into menopause several months before the transplant as the result of previous chemotherapy, while in the remaining patients ovarian cycles disappeared after the conditioning regimens. Only rare cases (<5%) of young women transplanted under 21 years, one of which had undergone an allo-HSCT for aplastic anemia and therefore had not received antiblastic agents in pretransplant period, experienced spontaneous recovery of ovarian function with regular menstrual cycles after 10–18 months of amenorrhea [26, 27].

**Figure 1:**
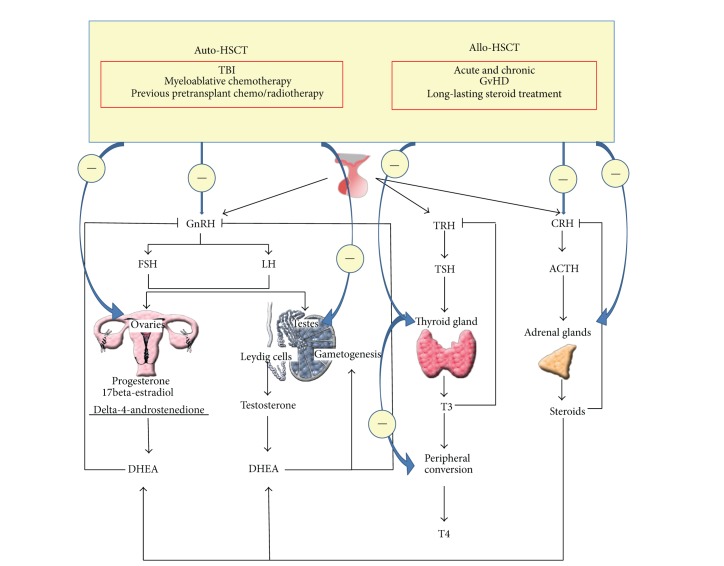
Main endocrine abnormalities after hematopoietic stem cell transplantation. Auto-HSCT and allo-HSCT: autologous- and allogeneic-hematopoietic stem cell transplantation; GnRH: gonadotropin releasing hormone; FSH: follicle-stimulating hormone; LH: luteinizing hormone; DHEA: dehydroepiandrosterone; TBI: total body irradiation; cGvHD: chronic graft-versus-host disease; ACTH: adrenocorticotropic hormone; CRH: corticotropin-releasing hormone; T4: thyroxine; T3: triiodothyronine; TSH: thyroid-stimulating hormone; TRH: thyrotropin releasing hormone. Symbol (−) means inhibition.

Although cycles recovered more frequently in auto-HSCT women, the difference when compared with the allo-HSCT group was not significant. However, recovery is not an early event and may occur long time after HSCT. Statistical analysis of predictive factors in our cohort of women undergoing allo- or auto-HSCT in their reproductive period showed that none of the following factors was directly related to the probability of spontaneous recovery of ovarian function: the type of transplant, bone mass index, and previous use of alkylating agents and/or steroids. In addition, we found that autotransplanted women had ovarian volumes similar to those observed in women after a corresponding interval from the physiological menopause, whereas the ovarian volumes of allo-HSCT were lower, suggesting greater damage to the ovarian tissue (Figure 1) [11].

Concerning hormonal pattern, serum 17beta-estradiol and Delta-4-androstenedione levels in our cohort of allo-HSCT patients were significantly decreased. In the group of allotransplanted women, signs of toxic effects on the ovaries were much more severe in those women affected by cGVHD. In contrast, in autotransplanted patients, only serum 17beta-estradiol levels were decreased, while Delta-4-androstenedione levels were found normal 12 ± 24 months after transplantation [11].

We have also documented reduced values of circulating androgens due to ovarian damage and adrenal suppression caused by immunosuppressive treatments, especially in women with cGVHD. Ovarian contribution to lower serum androgens was suggested by the correlation between ovarian volume and patients estradiol and androgen levels, although the lower dehydroepiandrosterone sulphate (DHEA-S) values observed in patients with cGVHD are also to be related to the prolonged use of corticosteroids [11, 26, 27].

A premature menopause for its clinical and psychological implications needs replacement treatment. The hormone replacement therapy (HRT) should be started after the complete hematologic recovery after transplantation, but it may be contraindicated for longer periods in women suffering from severe chronic liver GVHD. Indeed, estroprogestin may further worsen liver damage already caused by chronic GVHD [28, 29]. Moreover, HRT may not be fully effective if there is a simultaneous gastrointestinal or skin GVHD that interferes with the drug absorption; this was documented in 30% of women in a previous study [26]. In our experience, the cyclical sequential combination of estradiol (2 mg daily) plus dydrogesterone (10 mg for 14 days/month) was associated with excellent compliance, due to its simple administration and few adverse effects, allowing achieving a dramatic improvement of vasomotor, urogenital, and psychological symptoms mediated by estrogen deficiency. A withdrawal of hormone therapy for a period of 2 to 3 months per year together with monitoring of reproductive axis function is also suggested [27]. In fact, ultrasonographic evidence of ovarian follicles is often associated with a likelihood of cycles recovery, but there are no serum markers to predict the return of ovarian function in these patients. In our cohort of transplanted women, the cyclical sequential combination of estradiol (2 mg daily) plus dydrogesterone (10 mg for 14 days/month) was associated with excellent compliance because of its simple administration regimen and good safety and tolerability profile. In addition, the observed rate of ovarian function recovery in our center was 7% in the allo-HSCT setting and 25% in auto-HSCT setting [26, 27]. All the pieces of information, that is, ovarian ultrasonographic findings, hormonal pattern, and the recovery rates of ovarian function, are aligned in providing an evidence of more severe ovarian damage in the allogeneic HSCT setting (Figure 1).

A serious complication that may occur in the early posttransplant is polymenorrhea. Given that this complication can occur even before the full hematological recovery, especially when the platelet count is still low, the control of bleeding may be really difficult to manage [30]. The commonly used treatment for this complication is the norethisterone acetate that has been documented to be a risk factor for the development of liver venoocclusive disease [31]. Recently, the use of gonadotropin releasing hormone analogues (aGnRH), exerting suppressive effects on the hypothalamic-pituitary-gonadal axis, has been reported as being able to prevent peritransplant vaginal bleeding, without interfering with the hemostatic balance or inducing liver toxicity [32]. However, the administration of aGnRH should be started at least one month before the conditioning regimen due to the initial mild stimulatory effect on the hypothalamic-pituitary-gonad axis [31]. Although it has been established that hypogonadism induced by aGnRH is capable of preventing peritransplant bleeding, there is still insufficient evidence that it can be efficacious in preventing ovarian damage due to the antiblastic treatments [33].

Although the skin, the liver, and the gastrointestinal system are the sites most frequently involved in cGVHD, virtually any organ or tissue can be involved. The genital tract of women has been documented to be a potential target for cGVHD; in fact, variable degrees of vulvovaginal lesions related to gynecological cGVHD were described in 25% of cases by ourselves and by a large recent multicentric Italian study including 213 women [34, 35]. Milder forms of cGVHD were characterized by an increased frequency of developing vulvovaginal infections and inflammation, while the more severe forms have been found responsible for vaginal and cervical stenosis and malformations of the internal and external genitalia and sometimes associated even with perineal involvement. In addition, women with cGVHD may develop intrauterine adhesions that should be closely monitored by pelvic exam and ultrasonography during the first months of HRT, to avoid unpleasant complications such as hematocolpometra [36–38].

Therefore, female recipients of HSCT, and in particular those after allo-HSCT, require early surveillance and long-term follow-up of hormonal pattern and gynecological apparatus by skilled health care givers.

### 2.2. Men

Both alkylating agents and irradiation exert gonadotoxic effect on germ cell epithelium and Leydig cells of testis, in either childhood or adulthood [5, 39–43]. Usually there is a good correlation between the degree of spermatogenesis impairment and the corresponding increase of follicle-stimulating hormone (FSH) levels in patients after auto- and allo-HSCT. A return to elevated FSH levels within the normal range has been occasionally observed in patients treated with high-dose chemotherapy and TBI single fraction of 7.5 Gy, suggesting in these cases an unexpected recovery of germline function [44]. By contrast, recovery of spermatogenesis has been described very rarely in adult transplanted patients conditioned with fractionated TBI [45]. Molassiotis et al. documented persistently increased levels of FSH and luteinizing hormone (LH) in the majority of transplanted males, regardless of use of TBI [46].

In our experience impaired spermatogenesis damage was observed in all transplanted patients who underwent seminal fluid analysis at 12 months, about 90% of them showing germinal aplasia with azoospermia [25]. The increase in FSH levels was found only in 85% of cases, indicating that spermatogenesis damage was not always associated with increased FSH levels. The finding of oligozoospermia/azoospermia in transplanted patients that is lacking a corresponding FSH increase may have several explanations: (1) a partial damage to the hypothalamic-pituitary axis mediated by chemotherapy/radiotherapy leading to impaired gonadotrophin release, or (2) a partial/complete arrest of spermatogenesis at the spermatid level and of a rarer partial/complete occlusion of the spermatic tract [47].

In our cohort of transplanted patients, radiotherapy was associated with finding of significantly higher levels of FSH; this can be suggestive of a greater testicular toxic effect in patients treated with irradiation of abdominal/pelvic area [27]. Similarly, a greater cumulative probability for injury of germinal cells was previously reported in patients treated with radiation therapy when compared to those treated with chemotherapy only [17, 25, 41, 48, 49]. In addition, lower sperm counts were observed in long-term survivors affected by cGVHD when compared to unaffected patients, suggesting some possible effects of this chronic posttransplant complication on gonadal status [39, 50]. For men, the age at the time of treatment seems to be less important than for women in terms of susceptibility to gonadal failure. Conversely, the underlying disease, the type of antiblastic drugs, and/or the duration of their administration, all, affect spermatogenesis damage in men [5, 17].

Leydig cells are less vulnerable than germinal epithelium of the testis to the gonadotoxic effects of HSCT and/or treatments received prior to HSCT [40]. In fact, decrease of serum testosterone is mostly transient and recovers weeks-to-months after grafting (Figure 1) [45, 46, 51].

In our experience, testosterone was reduced in about 30% of patients up to 1 year after HSCT. However, testosterone production was unaffected in our long-term survivors out of treatments and none of the subjects reported a regression of secondary sexual characteristics. On the other hand, all men evaluated during acute and chronic GVHD had low testosterone levels, likely due to an inhibitory effect of the immunosuppressive treatments on the hypothalamic-pituitary-gonadal axis [27]. In fact, glucocorticoids are able to inhibit GnRH release and consequently the whole reproductive axis function. Moreover, glucocorticoids may also suppress the adrenal source of androgens [52]. The role of cyclosporine-A treatment in inducing Leydig cell damage cannot be excluded as it is known to exert a severe gonadotoxic effect on testicles in long-term treatments [53, 54]. The effects of new immunosuppressive drugs on gonadal function such as tacrolimus and mycophenolate mofetil have not yet been investigated exhaustively.

As sexual steroid hormone decrease in men after HSCT is mostly mild and transient; testosterone replacement is rarely required. There is one case report describing the failure of preventing TBI-induced testicular germinal cell damage by aGnRH administration. Currently, the higher chance to achieve a conceivement after HSCT, especially after allo-HSCT, is through sperm cryopreservation [55].

### 2.3. Fertility

The majority of auto- and allo-transplanted patients, as above described, experience different degree of gonadal damage that is responsible for their infertility. The ability to preserve fertility in these transplanted patients, mostly young people, is the subject of ongoing studies worldwide [56]. In the last two decades, aGnRH has been used to reduce and prevent chemotherapy-induced ovarian damage in small cohorts of young women with various types of cancer, and it has been reported that some patients receiving aGnRH in conjunction with gonadotoxic chemotherapy were able to spontaneously recover ovulation and becoming pregnant [57]. However, the ability of aGnRH to protect the ovarian function after HSCT is still debated. Although there are isolated case reports and a study on 30 women undergoing HSCT for hematological malignancy showing no benefit from aGnRH therapy in preventing the germ cell damage after HSCT [33], a more recent study in a cohort of 47 young transplanted women has reported that aGnRH administration in conjunction with conditioning chemotherapy before HSCT may significantly decrease the rate of premature ovarian failure from 82% to 33% in patients with lymphoma but not in those with leukemia [58–60].

Sperms and embryos conceived by* in vitro *fertilization can be well cryopreserved, but oocyte banking, in the absence of a male partner, is difficult and currently is still an experimental procedure. Not only the conservation but also oocyte retrieval is technically complex. For satisfactory oocyte retrieval, women need ovarian hyperstimulation for several weeks before starting high-dose chemotherapy, monitoring the follicular growth by ultrasonography. Following these procedures, a harvesting of the follicles should be performed. However, all these procedures before HSCT are often difficult to carry out, given the severity of the underlying malignant disease and the urgency to start antiblastic treatments as soon as possible [59, 61].

Occasional successful pregnancies have been reported in recent decades, especially in women who underwent autografting [62]. Most of data regarding this issue are derived from two large surveys performed in Europe and the USA. In both studies, partners of male patients had uncomplicated pregnancies and delivered normal children, whereas women after allografting showed a high incidence of miscarriage, preterm labor, and low birth weight babies, indicating the difficulty in completing their pregnancy due to damage of both the ovaries and gynecological tract or urogynecological apparatus. The rates of congenital malformations, developmental delays, and malignant diseases in the offspring of HSCT recipients were not different from those reported in the general population [24, 63].

In our experience of a total of 125 long-term survivors after allo-HSCT, only two pregnancies occurred: one was reported in a wife of a male patient who had received allograft 3 years earlier. One woman delivered two healthy twins 5 years after allo-SCT with hormonally fully assisted pregnancy obtained with oocytes donated by a sister [47].

Relapse of leukemia during or after pregnancy has been reported in various case reports. However, it cannot be excluded that these women have relapsed independently from pregnancy, and it is uncertain whether the natural history of the primary hematological disease can be influenced by pregnancy. Since the relationship between underlying diseases and pregnancy is still unclear, Salooja et al. recommended that pregnancy would be delayed by at least 2 years after HSCT [63].

## 3. Hypothalamus-Pituitary-Adrenal Axis

Secondary adrenal insufficiency due to the suppression of the hypothalamic-pituitary-adrenal axis is mainly related to the duration and cumulative dose of corticosteroid treatments received after HSCT. Only the initial report by Sanders et al. described high incidence of long-lasting adrenocortical insufficiency following TBI-based conditioning allo-HSCT [64], but subsequent studies have rarely documented a permanent reduction of plasma cortisol levels after transplant [26, 65]. More recently acute adrenal insufficiency after TBI-based conditioning regimens has been reported in children [66, 67].

In agreement with a previous hypothesis suggesting a relative resistance to irradiation of the adrenal tissue [4], in our cohort of nonirradiated allo-transplanted recipients, the onset of adrenal insufficiency was always related to the duration (more than 100 days) and cumulative dose (greater than 10 gr/m^2^) of corticosteroid treatment. However, though all our patients who developed a secondary adrenal insufficiency (a total of about 20% of cases in the auto- and allo-setting) were under treatment with corticosteroids, all of them having exceeded the cumulative dose and duration treatment above reported, we did not observe adrenal insufficiency in some age- and disease-matched patients which had assumed similar cumulative doses of steroids for similar periods, suggesting a variable-individual sensitivity of the hypothalamic-pituitary-adrenal axis to exogenous suppression. In our experience, corticoadrenal failure recovered in all patients after about 3–12 months of short acting steroid substitution therapy [26, 27]. Patients with chronic GVHD, in whom corticosteroid treatment is suddenly withdrawn because of the onset of serious infections, are at a high risk of developing an acute adrenal crisis that can further worsen their already compromised clinical condition [68–72]. As a stimulation test should always be carried out to rule out even a slight degree of dysfunction of the hypothalamic-pituitary-adrenal axis after high dose and/or chronic steroid treatment, the prevalence of nonovert adrenal insufficiency is likely an underestimated condition unless specifically investigated by endocrinologists. However, autoantibodies to the adrenal cortex (ACA) and to 21-hydroxylase assayed in our cohort of transplanted patients with adrenal insufficiency were absent (Figure 1) [27].

The use of replacement therapy with short acting steroids is recommended until the adrenal axis recovers in allo- and auto-HSCT recipients with a progressive reduction of the corticosteroid dose, to enable the adrenal axis to gradually recover [27, 73].

## 4. Hypothalamic-Pituitary-Insulin-Like Growth Factor-I Axis

Previous studies on GH secretion have documented that GH secreting pituitary cells are less vulnerable to irradiation damage in adulthood than in childhood. Littley et al. described a normal GH peak response to GH stimulation, 17–55 months after TBI-based conditioning regimen allo-HSCT [45]. Indeed, Kauppila et al. documented normal insulin-like growth factor-1 (IGF-1) levels in all transplanted patients, 20% of them showing impaired response to growth hormone-releasing hormone (GHRH) stimulation after TBI-based conditioning regimen allo-HSCT (<5 mcg/L with GHRH administration) [74]. In addition, decreased growth and GH secretion have previously been reported after cranial irradiation [75]. A less significant height reduction occurred after TBI [76–78].

Contradictory results were reported in children transplanted for leukemia after BU/CY conditioning regimen. Sanders showed a significant incidence of GH deficiency [79, 80], whereas Wingard et al. have documented a similar growth rate after BU/CY and CY/TBI in the first two years after HSCT [81]. Conversely, several other studies did not find any significant growth impairment up to 5 years after BU/CY-conditioning for allo-HSCT [82–86]. However, definitive conclusions about the conditioning BU/CY effect on the growth require further study.

Sanders et al. also found that cGVHD may result in impaired growth [64]. Glucocorticoids representing, alone and in combination with other immunosuppressive drugs, the first line therapy for cGVHD treatment exert acute growth-suppressive effects; however, decreased growth rates were also described in cGVHD patients treated without steroids, suggesting that immune system derangement underlying cGVHD physiopathology may contribute to their growth impairment [82]. Known risk factors for GH deficiency after transplant are summarized in Table 2.

Hypothalamus-pituitary-IGF-I axis function has not been systematically investigated in adult allo- and auto-HSCT recipients. In our cohort of allo-HSCT conditioned with BU/CY, we have found that, although the GH profile showed levels within the normal range in all subjects, IGF-I levels were lower in 38% of allo-HSCT recipients affected by cGVHD, whereas IGF-1 resulted in the normal range in only 7% of subjects cGVHD-free [26]. This finding may partially explain previous observations by Sanders et al. suggesting that cGVHD represents a condition of multiorgan injury associated with lower body mass index (BMI) [64]. However, the hypothesis of a possible influence of the general health conditions on the GH-IGF axis is also supported by le Roith et al. and Kami et al., who investigated the effects of complications after allo-HSCT conditioned with chemotherapy alone on the growth; they documented that children with any type of complications, especially those including starvation and malnutrition, showed decreased growth rates compared with normal growth of children without posttransplant complications [87, 88].

Similarly to allo-HSCT, we and others have reported that auto-HSCT recipients show serum IGF-I levels below the age-reference values (about 56% of patients in our cohort) within 3 months after transplant, with a subsequent return to normal range in about 20% of cases, while remaining low in the other 38% of cases up to 1 year after transplant. However, since IGF-I deficiency is often transient and less frequently persistent, the use of replacement therapy is not currently recommended. On the other hand, the follow-up of IGF-I levels is suggested after the recovery from transplant and adequate period from corticosteroid withdrawal [27].

## 5. Hypothalamus-Pituitary-Thyroid Axis

Frequent overt or subclinical thyroid dysfunctions, including persistent low T3 syndrome, chronic thyroiditis, subclinical hypo- or hyperthyroidism, and neoplastic transformation, have been described after allo-HSCT [7, 26, 88, 89] and more rarely after auto-HSCT [17, 27, 90].

Permanent or transitory hypothyroidism following TBI or BU-CY regimens, occurring also years after allo-HSCT, has been reported in both children and adults [74, 75, 91, 92]. The incidence of thyroid dysfunctions after fractionated TBI (15-16%) [63, 72, 93] is significantly lower than after single-dose TBI (46–48%) [6, 94–96]. However, the long-term effects of TBI-induced thyroid dysfunctions are still unknown as well as its timing and peak incidence [97]. Subclinical hypothyroidism, characterized by mildly elevated TSH levels with thyroid hormone within the normal range, has been frequently reported after TBI doses of 10–12 Gy, with overt hypothyroidism rarely documented [65, 91, 94, 95]. Al-Fiar et al. have also described a gradual increase in the incidence of hypothyroidism with increasing TBI doses. Subclinical hypothyroidism, within 2 years after allo-HSCT, was also documented after BU-CY conditioning regimen with an incidence of 11% versus 16.7% after 12 Gy TBI [98]. Similar frequency was reported by Toubert et al. in a cohort of patients (14%) not receiving TBI-based conditioning regimen [7].

“Low T3 syndrome” (normal FT4 and TSH and FT3 below ranges of normality) has been reported by Toubert et al. in 48% of patients at 3 months, falling to 19% at 14 months after allo-SCT, whereas Schulte et al. documented this syndrome in 100% of patients at day 14 after allo-HSCT, claiming that its persistence up to day 28 after transplant was associated with a higher probability of fatal outcome [7, 99]. Indeed, although the availability of sensitive and specific assays for the evaluation of subclinical hypothyroidism has now replaced the use of thyrotropin releasing hormone (TRH) test, Siegert et al. reported an exaggerated TSH response to TRH, after a mean of 3.2 years following transplant, in 35% of allo-SCT recipients showing also normal values of TSH [72, 100].

In our experience, subclinical hypothyroidism was found also later after allo-SCT, up to 5 years after allo-HSCT. Substitutive treatment with levothyroxine was given to all patients [26, 90]. In addition, we have found persistence of the “low T3 syndrome” in patients with chronic extensive GVHD, even 12–48 months after allo-HSCT, likely due to decreased extrathyroidal conversion of thyroxin to 3,5,3′ triiodothyronine induced by both chronic disease and glucocorticoid therapy [101, 102]. However, in our experience, low T3 syndrome did not represent a negative prognostic factor for the general outcome.

In the auto-HSCT setting, we detected subclinical hypothyroidism in 9% and 12% of patients at 3 months and at 12 months, respectively. The “low T3 syndrome” was documented in about 30% of patients at 3 months after auto-HSCT, whereas no one showed this syndrome at 12 months. As expected, the incidence of hypothyroidism was higher in patients previously treated (15–36 months earlier) with neck/thoracic radiotherapy than in untreated patients (50% versus 1.3%, resp.) [27, 90]. Transient “low T3 syndrome” may partly be the result of adverse nutrition and metabolic conditions that may persist for several months after transplantation, particularly in the allogeneic setting, and disappear thereafter; indeed, corticosteroids and antiblastic treatments may also contribute to this thyroid dysfunction, in particular in autologous setting [99]. The “low T3 syndrome” was usually asymptomatic, especially in auto-HSCT recipients, and did not require any treatment. Nevertheless, these patients should be monitored every 3 months until their endocrine values do not return into the normal range [103]. However, none of our allo- or auto-HSCT patients developed overt hypothyroidism; the possible explanation can consist in the fact that no one did receive TBI (Figure 1) [27, 90].

Increased frequency of transient subclinical hyperthyroidism (normal FT3 and FT4 levels and TSH values below the normal range) has been reported early after allo-HSCT (peak incidence, about 100 days), mainly within the period of immunologic reconstitution suggesting that the major pathogenetic factor of thyroid damage is the immune system derangement occurring within the first 6 months after transplant [26, 88, 90]. We diagnosed transient subclinical hyperthyroidism in 15% of patients 12–18 months after allo-HSCT, in agreement with data from a larger longitudinal study by Kami et al. (12.3%) [88]. A similar disorder may also occur after auto-HSCT; however, in auto-HSCT recipients the transient subclinical hyperthyroidism was documented to be less severe, perhaps due to a milder degree of immune system derangement occurring in the auto-HSCT setting. Posttransplant thyroid ultrasound showed a nonhomogeneous hypoechoic pattern in 30% of patients with subclinical hyperthyroidism and was sometimes associated with a mild increase in autoantibodies, suggesting chronic autoimmune thyroiditis [104–106].

On the other hand, biochemical and ultrasound evidence of chronic thyroiditis associated with normal thyroid function has been documented in patients assessed also 2–10 years after transplant. Discrepancies between functional and ultrasound results and the appearance or absence of thyroid autoantibodies, particularly in allo-HSCT recipients, may be related to the particular immunological conditions of these patients as well as to immunologic effects of immunosuppressive therapies. Transient hyperthyroidism was usually nonsymptomatic, especially in auto-HSCT recipients, and did not require any treatment [26, 27, 90]. However, both transplanted patients with transient hyperthyroidism and those with evidence of thyroiditis and normal thyroid function for their risk of developing hypothyroidism should be monitored every 3 to 4 months after transplant until their endocrine parameters are normalized [107, 108]. Known risk factors for thyroid dysfunction are summarized in Table 3.

Only a few studies have investigated the development of thyroid carcinoma in long-term survivors after HSCT [1, 109–114]. Increased incidences of secondary follicular and papillar thyroid carcinoma have been recently documented after HSCT recipients in the largest retrospective multicenter study (68.936 patients receiving allo- or auto-HSCT) that was carried out on this specific issue by the European Group for Blood and Marrow Transplantation (EBMT) registry [109]. In this large cohort of transplanted patients, a higher risk of secondary thyroid carcinoma (32 cases) has been documented compared to the general European population, with a ratio of observed to expected cases of 3.26. Median interval between HSCT and the diagnosis of secondary thyroid carcinoma was 8.5 years, similar only to that observed in patients with Hodgkin's disease [115]. Statistically significant risk factors for secondary thyroid carcinoma documented in this population were younger age (<10 years), TBI, dose of TBI, female sex, and chronic GVHD [109].

As there are several evidences that TSH increase may be a risk factor for thyroid tumorigenesis, generally the patients with subclinical hypothyroidism are treated with a substitutive levothyroxin dose in order to normalize TSH levels [116]. However, in the EBMT cohort of HSCT recipients developing thyroid carcinoma reported above, thyroid function abnormalities (serum TSH and thyroxin levels and thyroid specific antibodies) were found in only few cases, suggesting that thyroid laboratory tests do not help to suspect and diagnose the thyroid carcinoma in HSCT patients [109]. With the increasing use of ultrasound scans and fine-needle aspiration biopsy (FNAB), thyroid carcinoma tends to be diagnosed very accurately at early stages. Recently, Vivanco et al. reported an incidence of 8% for thyroid cancer after ultrasound examination in transplanted patients receiving TBI-based conditioning regimen HSCT during childhood, claiming the need to use for thyroid investigation in transplanted patients not only functional tests but also ultrasound scans, followed by FNAB of thyroid nodules when required, every 1–3 years after transplant [114].

Periodical yearly monitoring of thyroid function and morphology is recommended in HSCT recipients.

## 6. Conclusions

Autologous- (auto-) as well as allogeneic- (allo-) hematopoietic stem cell transplantation (HSCT) is nowadays an essential part of treatment strategy of several malignant and nonmalignant hematologic diseases as well as some nonhematologic diseases including inherited metabolic disorders. Often this is lifesaving therapeutic intervention. Over the past four decades, the transplant-related mortality progressively decreased, thanks to the development of less toxic pretransplant conditioning regimens and the improvement of prophylaxis and therapy of infections and GVHD as well as of other supportive care. These achievements resulted in parallel increase in the numbers of long-term HSCT survivors, a proportion of which is affected by early and late HSCT complications that warrant an appropriate management as they may significantly worsen the quality of life and cause long-term morbidity and mortality. Indeed, currently estimated increase in morbidity of long-term survivors after transplantation is five- to ten-fold versus matched general population over 30 years of observation and the estimated increase of mortality is about 30% in the same time frame. Both morbidity and mortality seem to be higher in the allogeneic setting. This is the reason why ever greater attention by physicians is now focused on how to better prevent, detect, and treat early and late complications and effects of HSCT.

The relatively high proportion of growing cells contained in the endocrine glands realize why early and late endocrine complications, varying from minimal subclinical symptoms to life-threatening disorders, are among the most common complications observed in survivors after auto- and allo-HSCT.

Gonadal failure has been found in approximately two-thirds of auto- and allo-HSCT patients, 90–99% of women and 60–90% of men, in both prevalently related to TBI- and high dose chemotherapy-based conditioning regimens. Impairments of hypothalamus-pituitary-GH/IGF-I and adrenal axis have been more frequently documented later and occurred in approximately 10% and 40–50% of patients, respectively, being related to antiblastic treatments, immune system derangement, and immunosuppressive treatments. Overt or subclinical thyroid dysfunctions, including persistent low T3 syndrome, chronic thyroiditis, subclinical hypo- or hyperthyroidism, and thyroid carcinoma, occurring in about 30% of HSCT recipients prevalently as late events may persist for many years, more frequently after allo-HSCT than auto-HSCT. Although it has been well established that TBI may predispose to thyroid disorders and neoplastic transformation, high-dose antiblastic treatments, alkylants agents, corticosteroid and acute and, especially, chronic GVHD development may also contribute to thyroid dysfunctions after transplant.

The underlying diseases, previous pretransplant therapies, the age at HSCT, TBI, and its dose and administration schedule, and posttransplant development of acute and chronic GVHD requiring prolonged high doses of steroids are the main risk factors for endocrine complications after HSCT.

Since the early identification of endocrine complications can greatly improve the quality of life of long-term survivors after HSCT and as it can be difficult for physicians who operate outside of specialized centers, it is mandatory to set up a multidisciplinary program consisting of hematologists, endocrinologists, and primary care physicians in order to decide the most appropriate investigating timing, prevention, diagnosis, and monitoring of multiple early and late endocrine disorders after auto- and allo-SCT. Personalized approach to each patient is preferable, including identification of endocrine disorders requiring treatment and those which need to be followed up.

## Figures and Tables

**Table 1:** Risk factors for gonadal damage.

	Risk factor degree
Patient relating factor	
Pubertal stage	Postpuberal >prepuberal
Age of HSCT in women	>30 years
Sex	F > M
Underlying disease	ALL, lymphomas
Treatment relating factor	
Type of radiotherapy	TBI, pelvic, inverted Y, fractionated doses
Chemotherapy	Alkylating agents > other chemotherapy
Type of transplant	Previous TBI Allo-HSCT >auto-HSCT
HSCT complications	Presence of GVHD

ALL: acute lymphoblastic leukemia; allo: allogeneic; auto: autologous; GVHD: graft-versus-host disease; HSCT: hematopoietic stem cell transplant; F: female; M: male; TBI: “total body” irradiation.

Symbol (>) means more than.

**Table 2:** Risk factors for GH deficiency.

	Risk factor degree
Patient relating factors	Pediatric age >adult age
Treatment relating factors	Radiotherapy >chemotherapy
	Single dose >fractioned dose TBI
	Intrathecal chemotherapy

TBI: “total body” irradiation. Symbol (>) means more than.

**Table 3:** Risk factors for thyroid function.

Disease	Risk factor degree	Timing from HSCT
Hypothyroidism	Radiotherapy to the neck >TBI	Late effect (years from HSCT)
	Single dose of TBI >fractionated TBI	
	Allo-HSCT >auto-HSCT	
	Chronic GVHD	

Subclinical hyperthyroidism	Allo-HSCT	Early effect (within 12 months after HSCT)

Low T3 syndrome	Infections Nutritional status Chronic GVHD	Variable up to years after HSCT
	Immunosuppressive therapies	

Thyroid carcinoma	Radiochemotherapy >chemotherapy	Late effect (years after HSCT)
	Pediatric age >adult age	

Allo: allogeneic; auto: autologous; GVHD: graft-versus-host disease; HSCT: hematopoietic stem cell transplant; TBI: “total body” irradiation.

Symbol (>) means more than.

## References

[1] Kolb HJ, Socié G, Duell T (1999). Malignant neoplasms in long-term survivors of bone marrow transplantation. Late effects working party of the European cooperative group for blood
and marrow transplantation and the European late effect project group. *Annals of Internal Medicine*.

[2] Horowitz M, Thomas ED, Blume KG, Forman SJ (1998). Uses and growth of hematopoetic cell transplantation. *Hematoipoietic Cell Transplantation*.

[3] Roziakova L, Mladosievicova B (2010). Endocrine late effects after hematopoietic stem cell transplantation. *Oncology Research*.

[4] Shalet SM, Didi M, Ogilvy-Stuart AL, Schulga J, Donaldson MDC (1995). Growth and endocrine functiom after bone marrow transplantation. *Clinical Endocrinology*.

[5] Brennan BMD, Shalet SM (2002). Endocrine late effects after bone marrow transplant. *British Journal of Haematology*.

[6] Thomas BC, Stanhope R, Plowman PN, Leiper AD (1993). Endocrine function following single fraction and fractionated total body irradiation for bone marrow transplantation in childhood. *Acta Endocrinologica*.

[7] Toubert M-E, Socié G, Gluckman E (1997). Short- and long-term follow-up of thyroid dysfunction after allogeneic bone marrow transplantation without the use of preparative total body irradiation. *British Journal of Haematology*.

[8] Galimberti M, de Sanctis V, Lucarelli G (1991). Endocrine function after bone marrow transplantation for Thalassemia. *Bone Marrow Transplantation*.

[9] Jadus MR, Wepsic HT (1992). The role of cytokines in graft-versus-host reactions and disease. *Bone Marrow Transplantation*.

[10] Sullivan KM, Agura E, Anasetti C (1991). Chronic graft-versus-host disease and other late complications of bone marrow transplantation. *Seminars in Hematology*.

[11] Tauchmanovà L, Selleri C, de Rosa G (2003). Gonadal status in reproductive age women after haematopoietic stem cell transplantation for haematological malignancies. *Human Reproduction*.

[12] Goodwin PJ, Ennis M, Pritchard KI, Trudeau M, Hood N (1999). Risk of menopause during the first year after breast cancer diagnosis. *Journal of Clinical Oncology*.

[13] Apperley JF, Apperley J, Gluckman E, Gratwohl A, Craddock C (1998). Late complications of transplants in adults. Fertility after high dose therapy. *Blood and Marrow Transplantation (the European School of Haematology Handbook 1998)*.

[14] Schubert MA, Sullivan KM, Schubert MM (1990). Gynecological abnormalities following allogeneic bone marrow transplantation. *Bone Marrow Transplantation*.

[15] Liu J, Malhotra R, Voltarelli J (2008). Ovarian recovery after stem cell transplantation. *Bone Marrow Transplantation*.

[16] Schimmer AD, Quatermain M, Imrie K (1998). Ovarian function after autologous bone marrow transplantation. *Journal of Clinical Oncology*.

[17] Keilholtz U, Max R, Scheibenbogen C, Wüster Ch, Körbling M, Haas R (1997). Endocrine function and bone metabolism 5 years after autologous bone marrow/blood-derived progenitor cell transplantation. *Cancer*.

[18] Sanders JE, Buckner CD, Amos D (1988). Ovarian function following marrow transplantation for aplastic anemia or leukemia. *Journal of Clinical Oncology*.

[19] Apperley JF, Reddy N (1995). Mechanism and management of treatment-related gonadal failure in recipients of high dose chemoradiotherapy. *Blood Reviews*.

[20] Wallace WHB, Shalet SM, Crowne EC, Morris-Jones PH, Gattamaneni HR (1989). Ovarian failure following abdominal irradiation in childhood: natural history and prognosis. *Clinical Oncology*.

[21] Chabner BA, Allegra CJ, Curt A, Hardman JG, Limbird LE (1996). Antineoplastic agents. *Goodman & Gilman’s The Pharmacological Basis of Therapeutics*.

[22] Warne GL, Fairley KF, Hobbs JB, Martin FIR (1973). Cyclophosphamide induced ovarian failure. *The New England Journal of Medicine*.

[23] Sanders JE, Hawley J, Levy W (1996). Pregnancies following high-dose cyclophosphamide with or without high-dose busulfan or total-body irradiation and bone marrow transplantation. *Blood*.

[24] Cho W-K, Lee J-W, Chung NG (2011). Primary ovarian dysfunction after hematopoietic stem cell transplantation during childhood: Busulfan-based conditioning is a major concern. *Journal of Pediatric Endocrinology and Metabolism*.

[25] Chatterjee R, Mills W, Katz M, McGarrigle HH, Goldstone AH (1994). Germ cell failure and Leydig cell insufficiency in post-pubertal males after autologous bone marrow transplantation with BEAM for lymphoma. *Bone Marrow Transplantation*.

[26] Tauchmanovà L, Selleri C, de Rosa G (2002). High prevalence of endocrine dysfunction in long-term survivors after allogeneic bone marrow transplantation for hematologic diseases. *Cancer*.

[27] Tauchmanovà L, Selleri C, de Rosa G (2005). Endocrine disorders during the first year after autologous stem-cell transplant. *The American Journal of Medicine*.

[28] Choidi S, Spinelli S, Cohen A (1991). Cyclic sex hormone replacement therapy in women undergoing allogeneic bone marrow transplantation: aims and results. *Bone Marrow Transplantation*.

[29] Savani BN, Griffith ML, Jagasia S, Lee SJ (2011). How I treat late effects in adults after allogeneic stem cell transplantation. *Blood*.

[30] Laufer MR, Townsend NL, Parsons KE (1997). Inducing amenorrhea during bone marrow transplantation: a pilot study of leuprolide acetate. *Journal of Reproductive Medicine for the Obstetrician and Gynecologist*.

[31] Hägglund H, Remberger M, Klaesson S, Lönnqvist B, Ljungman P, Ringdén O (1998). Norethisterone treatment, a major risk-factor for veno-occlusive disease in the liver after allogeneic bone marrow transplantation. *Blood*.

[32] Sica S, Salutari P, Chiusolo P (1999). Hormonal therapy after stem cell transplantation and the risk of veno- occlusive disease. *Blood*.

[33] Chiusolo P, Salutari P, Sica S (1998). Luteinizing hormone-releasing hormone analogue: leuprorelin acetate for the prevention of menstrual bleeding in premenopausal women undergoing stem cell transplantation. *Bone Marrow Transplantation*.

[34] Tauchmanovà L, Selleri C, de Rosa G (2007). Estrogen-progestin therapy in women after stem cell transplant: our experience and literature review. *Menopause*.

[35] Spinelli S, Chiodi S, Costantini S (2003). Female genital tract graft-versus-host disease following allogeneic bone marrow transplantation. *Haematologica*.

[36] Tauchmanovà L, Selleri C, di Carlo C (2004). Estrogen-progestogen induced hematocolpometra following allogeneic stem cell transplant. *Gynecologic Oncology*.

[37] Anguenot J-L, Ibéchéole V, Helg C, Piacenza J-M, Dumps P, Bonnefoi H (2002). Vaginal stenosis with hematocolpometra, complicating chronic graft versus host disease. *European Journal of Obstetrics Gynecology and Reproductive Biology*.

[38] Syrjala KL, Roth-Roemer SL, Abrams JR (1998). Prevalence and predictors of sexual dysfunction in long-term survivors of marrow transplantation. *Journal of Clinical Oncology*.

[39] Grigg AP, McLachlan R, Zajac J, Szer J (2000). Reproductive status in long-term bone marrow transplant survivors receiving busulfan-cyclophosphamide (120 mg/kg). *Bone Marrow Transplantation*.

[40] Shalet SM, Sheaves R, Jenkins P, Wass JAH (1994). Cancer therapy and gonadal dysfunction. *Clinical Endocrine Oncology*.

[41] Mertens AC, Ramsay NKC, Kouris S, Neglia JP (1998). Patterns of gonadal dysfunction following bone marrow transplantation. *Bone Marrow Transplantation*.

[42] Harris E, Mahendra P, McGarrigle HH, Linch DC, Chatterjee R (2001). Gynaecomastia with hypergonadotrophic hypogonadism and Leydig cell insuffiency in recipients of high-dose chemotherapy or chemo-radiotherapy. *Bone Marrow Transplantation*.

[43] Anserini P, Chiodi S, Spinelli S (2002). Gonadal function post transplantation. Semen analysis following allogeneic bone marrow transplantation. Additional data for evidence-based counselling. *Bone Marrow Transplantation*.

[44] Sklar CA, Kim TH, Ramsay NKC (1984). Testicular function following bone marrow transplantation performed during or after puberty. *Cancer*.

[45] Littley MD, Shalet SM, Morgenstern GR, Deakin DP (1991). Endocrine and reproductive dysfunction following fractionated total body irradiation in adults. *Quarterly Journal of Medicine*.

[46] Molassiotis A, van den Akker OBA, Milligan DW, Boughton BJ (1995). Gonadal function and psychosexual adjustment in male long-term survivors of bone marrow transplantation. *Bone Marrow Transplantation*.

[47] Tauchmanovà L, Alviggi C, Foresta C (2007). Cryptozoospermia with normal testicular function after allogeneic stem cell transplantation: a case report. *Human Reproduction*.

[48] Walters MC, Hardy K, Edwards S (2010). Pulmonary, gonadal, and central nervous system status after bone marrow transplantation for sickle cell disease. *Biology of Blood and Marrow Transplantation*.

[49] Kyriacou C, Kottaridis PD, Eliahoo J (2003). Germ cell damage and Leydig cell insufficiency in recipients of nonmyeloablative transplantation for haematological malignancies. *Bone Marrow Transplantation*.

[50] Rovó A, Tichelli A, Passweg JR (2006). Spermatogenesis in long-term survivors after allogeneic hematopoietic stem cell transplantation is associated with age, time interval since transplantation, and apparently absence of chronic GvHD. *Blood*.

[51] van Basten J-PA, van Driel MF, Hoekstra HJH, Sleijfer DT (2000). Erectile dysfunction with chemotherapy. *The Lancet*.

[52] Nieman LK, Ilias I (2005). Evaluation and treatment of Cushing’s syndrome. *The American Journal of Medicine*.

[53] Handelsman DJ, Mc Dowell IF, Caterson ID, Tiller DJ, Hall BM, Turtle JR (1984). Testicular function after renal transplantation: comparison of Cyclosporin A with azathioprine and prednisone combination regimes. *Clinical Nephrology*.

[54] Schimmer AD, Ali V, Stewart AK, Imrie K, Keating A (2001). Male sexual function after autologous blood or marrow transplantation. *Biology of Blood and Marrow Transplantation*.

[55] Chatterjee R, Kottaridis PD, McGarrigle HH, Papatryphonos A, Goldstone AH (2001). Hypogonadotrophism fails to prevent severe testicular damage induced by total body irradiation in a patient with beta-thalassaemia major and acute lymphoblastic leukaemia. *Bone Marrow Transplantation*.

[56] Nakayama K, Liu P, Detry M (2009). Receiving information on fertility- and menopause-related treatment effects among women who undergo hematopoietic stem cell transplantation: changes in perceived importance over time. *Biology of Blood and Marrow Transplantation*.

[57] Chatterjee R, Kottaridis PD (2002). Treatment of gonadal damage in recipients of allogeneic or autologous transplantation for haematological malignancies. *Bone Marrow Transplantation*.

[58] Blumenfeld Z, Patel B, Leiba R, Zuckerman T (2012). Gonadotropin-releasing hormone agonist may minimize premature ovarian failure in young women undergoing autologous stem cell transplantation. *Fertility and Sterility*.

[59] Cheng YC, Takagi M, Milbourne A, Champlin RE, Ueno NT (2012). Phase ii study of gonadotropin-releasing hormone analog for ovarian function preservation in hematopoietic stem cell transplantation patients. *Oncologist*.

[60] Cornillon J, Decanter C, Couturier MA (2013). Management of endocrine dysfunctions after allogeneic hematopoietic stem cell transplantation: a report of the SFGM-TC on gonadal failure and fertility. *Pathologie Biologie*.

[61] Fabbri R, Porcu E, Marsella T, Rocchetta G, Venturoli S, Flamigni C (2001). Human oocyte cryopreservation: new perspectives regarding oocyte survival. *Human Reproduction*.

[62] Hershlag A, Schuster MW (2002). Return of fertility after autologous stem cell transplantation. *Fertility and Sterility*.

[63] Salooja N, Szydlo RM, Sociè G (2001). Pregnancy outcomes after peripheral blood or bone marrow transplantation: a retrospective survey. *The Lancet*.

[64] Sanders JE, Pritchard S, Mahoney P (1986). Growth and development following marrow transplantation for leukemia. *Blood*.

[65] Ogilvy-Stuart AL, Clark DJ, Wallace WHB (1992). Endocrine deficit after fractionated total body irradiation. *Archives of Disease in Childhood*.

[66] Al-Anazi KA, Nassar A, Elghazaly A, Bakr M, El-Tayeb KI, Chaudhri N (2010). Acute adrenal insufficiency induced by total body irradiation in a recipient of an allogeneic hematopoietic stem cell transplantation. *Cell & Tissue Transplantation & Therapy*.

[67] Savas-Erdeve S, Berberoglu M, Siklar Z (2011). Primary adrenal insufficiency in a child after busulfan and cyclophosphamide-based conditioning for hematopoietic stem cell transplantation. *Journal of Pediatric Endocrinology and Metabolism*.

[68] Glucksberg H, Storb R, Fefer A (1974). Clinical manifestations of graft versus host disease in human recipients of marrow from HL A matched sibling donors. *Transplantation*.

[69] Shulman HM, Sullivan KM, Weiden PL (1980). Chronic Graft-Versus-Host syndrome in man. A long-term clinicopathologic study of 20 Seattle patients. *The American Journal of Medicine*.

[70] Bostrom L, Ringden O, Jacobsen N, Zwaan F, Nilsson B (1990). A European multicenter study of chronic graft-versus-host disease. The role of cytomegalovirus serology in recipients and donors—acute graft-versus-host disease, and splenectomy. *Transplantation*.

[71] Kier P, Penner E, Bakos S (1990). Autoantibodies in chronic GVHD: high prevalence of antinucleolar antibodies. *Bone Marrow Transplantation*.

[72] Siegert W, Stemerowicz R, Hopf U (1992). Antimitochondrial antibodies in patients with chronic graft-versus-host disease. *Bone Marrow Transplantation*.

[73] Cornillon J, Vantyghem MC, Couturier MA (2013). Management of endocrine dysfunctions after allogeneic hematopoietic stem cell transplantation: a report of the SFGM-TC on adrenal insufficiency and osteoporosis. *Pathologie Biologie*.

[74] Kauppila M, Koskinen P, Irjala K, Remes K, Viikari J (1998). Long-term effects of allogeneic bone marrow transplantation (BMT) on pituitary, gonad, thyroid and adrenal function in adults. *Bone Marrow Transplantation*.

[75] Leiper AD, Stanhope R, Lau T (1987). The effect of total body irradiation and bone marrow transplantation during childhood and adolescence on growth and endocrine function. *British Journal of Haematology*.

[76] Cohen A, Rovelli A, Bakker B (1999). Final height of patients who underwent bone marrow transplantation for hematological disorders during childhood: a study by the working party for late effects-EBMT. *Blood*.

[77] Holm K, Nysom K, Rasmussen MH (1996). Growth, growth hormone and final height after BMT. Possible recovery of irradiation-induced growth hormone insufficiency. *Bone Marrow Transplantation*.

[78] Dvorak CC, Gracia CR, Sanders JE (2011). NCI, NHLBI/PBMTC first international conference on late effects after pediatric hematopoietic cell transplantation: endocrine challenges-thyroid dysfunction, growth impairment, bone health, & reproductive risks. *Biology of Blood and Marrow Transplantation*.

[79] Sanders JE (1991). The impact of marrow transplant preparative regimens on subsequent growth and development. *Seminars in Hematology*.

[80] Sanders JE (2008). Growth and development after hematopoietic cell transplant in children. *Bone Marrow Transplantation*.

[81] Wingard JR, Plotnick LP, Freemer CS (1992). Growth in children after bone marrow transplantation: Busulfan plus cyclophosphamide versus cyclophosphamide plus total body irradiation. *Blood*.

[82] Liesner RJ, Leiper AD, Hann IM, Chessells JM (1994). Late effects of intensive treatment for acute myeloid leukemia and myelodysplasia in childhood. *Journal of Clinical Oncology*.

[83] Giorgiani G, Bozzola M, Locatelli F (1995). Role of busulfan and total body irradiation on growth of prepubertal children receiving bone marrow transplantation and results of treatment with recombinant human growth hormone. *Blood*.

[84] Michel G, Socié G, Gebhard F (1997). Late effects of allogeneic bone marrow transplantation for children with acute myeloblastic leukemia in first complete remission: the impact of conditioning regimen without total-body irradiation—a report from the Societe Francaise de Greffe de Moelle. *Journal of Clinical Oncology*.

[85] Afify Z, Shaw PJ, Clavano-Harding A, Cowell CT (2000). Growth and endocrine function in children with acute myeloid leukaemia after bone marrow transplantation using busulfan/cyclophosphamide. *Bone Marrow Transplantation*.

[86] Felicetti F, Manicone R, Corrias A (2011). Endocrine late effects after total body irradiation in patients who received hematopoietic cell transplantation during childhood: a retrospective study from a single institution. *Journal of Cancer Research and Clinical Oncology*.

[87] le Roith D, Bondy C, Yakar S, Liu J-L, Butler A (2001). The somatomedin hypothesis: 2001. *Endocrine Reviews*.

[88] Kami M, Tanaka Y, Chiba S (2001). Thyroid function after bone marrow transplantation: possible association between immune-mediated thyrotoxicosis and hypothroidism. *Transplantation*.

[89] Benker G, Hermanns U, Mahmoud MK (1989). Allogenic bone marrow transplantation in adults: endocrine sequelae after 1–6 years. *Acta Endocrinologica*.

[90] Tauchmanovà L, Colao A, Selleri C, de Rosa G, Rotoli B (2006). Thyroid dysfunction after autologous hematopoietic stem cell transplant. *The American Journal of Medicine*.

[91] Katsanis E, Shapiro RS, Robison LL (1990). Thyroid dysfunction following bone marrow transplantation: long-term follow-up of 80 pediatric patients. *Bone Marrow Transplantation*.

[92] Curtillet C, Cuadras P, Dore E, Chambost H, Thuret I, Michel G (2004). Thyroid dysfunction after haematopoietic stem cell transplantation during childhood. *Archives de Pediatrie*.

[93] Lee V, Cheng PS, Chik KW, Wong GWK, Shing MMK, Li CK (2006). Autoimmune hypothyroidism after unrelated haematopoietic stem cell transplantation in children. *Journal of Pediatric Hematology/Oncology*.

[94] Cohen A, Apperley J, Gluckman E, Gratwohl A, Craddock C (2000). Late complications of transplants. Endocrinological complications. *Blood and Marrow Transplantation, the European School of Haematology Handbook 1998*.

[95] Gunasekaran U, Agarwal N, Jagasia MH, Jagasia SM (2012). Endocrine complications in long-term survivors after allogeneic stem cell transplant. *Seminars in Hematology*.

[96] Borgstrom B, Bolme P (1994). Thyroid function in children after allogeneic bone marrow transplantation. *Bone Marrow Transplantation*.

[97] Sanders JE, Hoffmeister PA, Woolfrey AE (2009). Thyroid function following hematopoietic cell transplantation in children: 30 years’ experience. *Blood*.

[98] Al-Fiar FZ, Colwill R, Lipton JH, Fyles G, Spaner D, Messner H (1997). Abnormal thyroid stimulating hormone (TSH) levels in adults following allogeneic bone marrow transplants. *Bone Marrow Transplantation*.

[99] Schulte C, Reinhardt W, Beelen D, Mann K, Schaefer U (1998). Low T3-syndrome and nutritional status as prognostic factors in patients undergoing bone marrow transplantation. *Bone Marrow Transplantation*.

[100] Somali M, Mpatakoias V, Avramides A (2005). Thyroid dysfunction in adult long-term survivors after hemapoeitic stem-cell transplantation (HSCT). *Hormone and Metabolic Research*.

[101] Chopra IJ, Williams DE, Orgiazzi J, Solomon DH (1975). Opposite effects of dexamethasone on serum concentrations of 3,3′,5′ triiodothyronine (reverse T3) and 3,3’,5 triiodothyronine (T3). *Journal of Clinical Endocrinology and Metabolism*.

[102] Chopra IJ, Huang TS, Beredo A (1985). Evidence for an inhibitor of extrathyroidal conversion of thyroxine to 3,5,3′-triiodothyronine in sera of patients with nonthyroidal illnesses. *Journal of Clinical Endocrinology and Metabolism*.

[103] Cornillon J, Vantyghem MC, Couturier MA (2013). Management of endocrine dysfunctions after allogeneic hematopoietic stem cell transplantation: a report of the SFGM-TC on dyslipidemia and thyroid disorders. *Pathologie Biologie*.

[104] Paydas S (2005). Autoimmune thyroid dysfunction after hematopoietic stem cell transplantation. *Bone Marrow Transplantation*.

[105] Au WY, Lie AK, Kung AW, Mak W, Kwong YL (2005). Thyrotoxic periodic paralysis after allogeneic haematopoietic stem cell transplantation. *British Journal of Haematology*.

[106] Au WY, Lie AKW, Kung AWC, Liang R, Hawkins BR, Kwong YL (2005). Autoimmune thyroid dysfunction after hematopoietic stem cell transplantation. *Bone Marrow Transplantation*.

[107] Feng Y-H, Su B-A, Lin C-Y, Huang W-T, Tsao C-J (2008). Hyperthyroidism as a latent complication of autologous hematopoietic stem cell transplantation. *International Journal of Hematology*.

[108] Hon C, Yau K, Chan EYT, Lee K-K, Au WY (2006). Graves’ ophthalmopathy after allogeneic stem cell transplantation. *Annals of Hematology*.

[109] Cohen A, Békássy AN, Gaiero A (2008). Endocrinological late complications after hematopoietic SCT in children. *Bone Marrow Transplantation*.

[110] Curtis RE, Rowlings PA, Deeg HJ (1997). Solid cancers after bone marrow transplantation. *The New England Journal of Medicine*.

[111] Bhatia S, Louie AD, Bhatia R (2001). Solid cancers after bone marrow transplantation. *Journal of Clinical Oncology*.

[112] Socié G, Curtis RE, Deeg HJ (2000). New malignant diseases after allogeneic marrow transplantation for childhood acute leukemia. *Journal of Clinical Oncology*.

[113] Pacini F, Vorontsova T, Molinaro E (1999). Thyroid consequences of the Chernobyl nuclear accident. *Acta Paediatrica, Supplement*.

[114] Vivanco M, Dalle JH, Alberti C (2012). Malignant and benign thyroid nodules after total body irradiation preceding hematopoietic cell transplantation during childhood. *European Journal of Endocrinology*.

[115] Sklar C, Whitton J, Mertens A (2000). Abnormalities of the thyroid in survivors of Hodgkin’s disease: data from the childhood cancer survivor study. *Journal of Clinical Endocrinology and Metabolism*.

[116] Rivas M, Santisteban P (2003). TSH-activated signaling pathways in thyroid tumorigenesis. *Molecular and Cellular Endocrinology*.

